# Hypertension screening, awareness, treatment, and control: a study of their prevalence and associated factors in a nationally representative sample from Nepal

**DOI:** 10.1080/16549716.2021.2000092

**Published:** 2022-02-08

**Authors:** Raja Ram Dhungana, Zeljko Pedisic, Meghnath Dhimal, Bihungum Bista, Maximilian de Courten

**Affiliations:** a Institute for Health and Sport, Victoria University, Melbourne, Australia; b Nepal Health Research Council, Kathmandu, Nepal; c Mitchell Institute for Education and Health Policy, Victoria University, Melbourne, Australia

**Keywords:** Hypertension, awareness, treatment, control, Nepal

## Abstract

**Background:**

The growing burden of hypertension is emerging as one of the major healthcare challenges in low- and middle-income countries (LMICs), such as Nepal. Given that they are struggling to deliver adequate health services, some LMICs have significant gaps in the cascade of hypertension care (including screening, awareness, treatment, and control). This results in uncontrolled hypertension, placing a high burden on both patients and healthcare providers.

**Objective:**

The objective of this study was to quantify the gaps in hypertension screening, awareness, treatment, and control in the Nepalese population.

**Methods:**

We used the data from a pooled sample of 9682 participants collected through two consecutive STEPwise approach to Surveillance (STEPS) surveys conducted in Nepal in 2013 and 2019. A multistage cluster sampling method was applied in the surveys, to select nationally representative samples of 15- to 69-year-old Nepalese individuals. Prevalence ratios were calculated using multivariable Poisson regression.

**Results:**

Among the hypertensive participants, the prevalence of hypertension screening was 65.9% (95% CI: 62.2, 69.5), the prevalence of hypertension awareness was 20% (95% CI: 18.1, 22.1), the prevalence of hypertension treatment was 10.3% (95% CI: 8.8, 12.0), and the prevalence of hypertension control was 3.8% (95% CI: 2.9, 4.9). The unmet need of hypertension treatment and control was highest amongst the poorest individuals, the participants from Lumbini and Sudurpaschim provinces, those who received treatment in public hospitals, the uninsured, and those under the age of 30 years.

**Conclusions:**

The gaps in the cascade of hypertension care in Nepal are large. These gaps are particularly pronounced among the poor, persons living in Lumbini and Sudurpaschim provinces, those who sought treatment in public hospitals, those who did not have health insurance, and young people. National- and local-level public health interventions are needed to improve hypertension screening, awareness, treatment, and control in Nepal.

## Background

According to recent estimates, hypertension is the biggest single contributor to death and disability globally, accounting for 10.4 million deaths a year in 2017 [1]. Around 45% of deaths due to heart disease and 51% of deaths due to stroke are attributable to hypertension [2]. Hypertension affects more than 20% of the world’s adult population [3,4]. Among these hypertensive individuals, 75% are from low-income and middle-income countries (LMICs). In LMICs, the prevalence of hypertension increased by 7.7% between 2000 and 2010 [5].

The increasing prevalence of hypertension is also a growing concern in Nepal. Surveys conducted in different parts of Nepal between 2011 and 2016 suggest that the prevalence of hypertension has increased over the past decade [6–9]. A recent systematic review found that the prevalence of hypertension in Nepal increased by 6% between 2000 and 2020 [10].

The prevalence of hypertension screening, awareness, treatment, and control are low in LMICs, indicating gaps in the cascade of hypertension care [5]. Among all hypertensives in LMICs in 2010, 37.9% were aware that they had high blood pressure, 29.0% were receiving treatment, and only 7.7% had controlled blood pressure [5]. The burden of untreated and uncontrolled hypertension was also found to be high in Nepal’s neighbouring countries (e.g. Bangladesh, India, and Pakistan) in a study conducted between 2003 and 2009, where 68.1% of hypertensive persons did not receive treatment, and 87.1% of hypertensive persons did not have optimal control of their blood pressure [11]. Some of the sub-national study results suggest that the prevalence of hypertension awareness, treatment, and control are also low in Nepal [8,12–14]. A secondary analysis of the Demographic and Health Survey (DHS) 2016 results demonstrated that 38% of hypertensive people in Nepal were aware of their high blood pressure status, while 18% of hypertensive people were taking antihypertensive medication [15].

Gaps in the cascade of hypertension care are disproportionately distributed across different socio-demographic groups. Studies have shown that hypertension control is significantly lower in younger South Asian individuals as compared with other age groups [11]. Similarly, women, poor members of society (lowest wealth quintile), individuals with low levels of education, and those living in rural settings were more likely to have untreated and uncontrolled high blood pressure or be unaware of their hypertension [11]. An Indian study observed that single, men, participants from rural areas, and individuals with lower household wealth had a poorer status at each step in the cascade of care process [16].

Quantifying the unmet need to provide care and understanding its distribution in each step of the care process is critical for the effective management of the disease. The cascade of care framework is commonly used to describe and track the sequential steps across the continuum of care in the treatment of infectious diseases, and particularly in the treatment of HIV, hepatitis C, and tuberculosis [17]. In HIV infection, the concept of continuum care ‘seek, test, treat, and retain’ emphasizes the importance of identifying and diagnosing conditions early on and subsequently linking these conditions to antiretroviral therapy [18]. In addition, applying this concept helps to quantify the unmet need for services, as it enables users to track the loss of a proportion of service users at a particular stage in the cascade of care [19]. Recently, the concept was applied to assess the gaps in detecting and treating people with diabetes and hypertension and retaining them in the care process [19–21]. Healthcare providers and other stakeholders can apply the framework to identify persons who are more likely to be unaware of their condition, who are aware but untreated, or who have received treatment but who have sub-optimally controlled blood pressure. This framework also allows to locate gaps in the hypertension control cascade and tailor interventions to those in at-risk population groups [21]. For example, if a large proportion of the population is unaware of their condition, blood pressure screening or other outreach services can be used effectively to identify persons with hypertension. Similarly, if the lack of treatment or control is the prevailing problem, the stakeholders will need to identify and tackle health systems, health providers, and individual level barriers, in order to effectively connect the patient with the hypertension management services and retain them in the process of care.

Several studies have reported the prevalence of hypertension in Nepal, including the two recent Nepalese STEPwise approach to Surveillance (STEPS) surveys [8,22,23]. However, these studies did not systematically investigate the gaps in hypertension care using the cascade of care framework. This study was, therefore, carried out to quantify the losses of the hypertensive participants which occurred at each step of hypertension care cascade, and to determine the distribution of screening, awareness, treatment, and control of hypertension across different population groups in Nepal.

## Methods

### Data source, study participants, and sampling

We analysed data from two STEPwise approach to Surveillance (STEPS) surveys conducted in Nepal in 2013 and 2019. Both were nationally representative surveys in which the multistage cluster sampling method was used to select a single individual with 15 to 69 years of age from each sampled household. STEPS 2013 collected data from 4200 respondents selected from 210 clusters between January and June 2013. STEPS 2019 collected data from 5593 individuals from 737 clusters between October 2018 and March 2019. The response rates were 98.6% in 2013 and 86.4% in 2019. Detailed information about the survey methodology for STEPS 2013 [22] and STEPS 2019 [23] have been described elsewhere. The available data from the two surveys were combined to form a single dataset that included information about 9682 participants.

### Data collection

The STEPS survey used the World Health Organization (WHO) NCD STEPS instrument, structured into STEP I, STEP II, and STEP III to measure the behavioural, anthropometric, and biological characteristics of the participants [22,23]. For our study purpose, we extracted the socio-demographic (age, gender, marital status, education, occupation, and province), socio-economic (wealth quintile), behavioural risk factors (smoking, alcohol consumption, fruit and vegetable intake, and physical activity), and cardiometabolic risk factor (high body mass index, diabetes, and high cholesterol) data from the survey. We also included the STEPS survey 2019 data on health providers and health insurance in the subsequent analysis.

### Outcome variables

The outcome variables were hypertension screening, awareness, treatment, and control, which are collectively defined as the cascade of hypertension care. To assess hypertension screening, the surveys asked if the individual participants had ever had their blood pressure measured by a doctor or another health worker. Participants were considered aware if they knew they had high blood pressure, which had to have been diagnosed by the doctor or another health worker. Hypertension treatment was defined as the use of any antihypertensive medication to lower blood pressure at the time of data collection. We considered that hypertension was controlled, if the participants had a systolic blood pressure below 140 mmHg and a diastolic blood pressure below 90 mmHg. The unmet need for the cascade of care was categorised as: unscreened, unaware, untreated, and uncontrolled hypertension. This need was assessed using the reciprocal values of screening, awareness, treatment, and control of hypertension, respectively.

The systolic and diastolic blood pressure was measured using a digital, automated blood pressure monitor (OMRON digital device, OMRON, Netherlands) with a medium-sized cuff. Before blood pressure measurements were taken, the survey data enumerators asked the participants to rest for 15 minutes, roll up their clothing over their arm, sit up straight and quietly, and keep their legs uncrossed. The enumerators recorded three systolic and diastolic blood pressure readings at five-minute intervals. We averaged the second and third readings to obtain the final blood pressure readings. Participants were considered as hypertensive, if they had systolic blood pressure ≥ 140 mm Hg and/or diastolic blood pressure ≥ 90 mm Hg or were taking anti-hypertensive medications as recommended by the Joint National Committee-VII [24].

### Explanatory variables

We used the pre-existing categories of age, gender, marital status, education, and occupation as defined by the survey. The 2019 survey was the first STEPS survey to record the household wealth index. This index was divided into quintiles, with the lowest quintile denoting the poorest subgroup. The 2019 survey was also the first one to collect data based on the new provincial system and data on health insurance.

The surveys followed the WHO standard international guidelines to collect data on behavioural, clinical, and metabolic risk factors. A detailed description of the data collection methods used is available elsewhere [22,23]. Briefly, survey data were collected on smoking, alcohol consumption; the frequency and amount of fruit and vegetable intake (using a food frequency questionnaire), and physical activity (using the Global Physical Activity Questionnaire – GPAQ). In addition, participants’ height and weight were measured, and blood samples were analysed to assess the fasting blood sugar and lipid levels.

‘Current smokers’ were considered as participants who had smoked tobacco at least once in the 30 days prior to the survey [25]. Alcohol users were considered those who had drunk at least one alcoholic drink in the 30 days prior to the survey [26]. Eating at least two servings of fruit and at least three serving of vegetables per day in a typical week was considered as a sufficient fruit and vegetable intake [27]. Sufficient physical activity was defined as the involvement in moderate and/or vigorous physical activity equivalent to ≥ 600 MET minutes/week [28]. The body mass index (BMI) was calculated as the weight (in kg) divided by the height (in meters) squared and categorised into < 25.0 kg/m^2^ (as not overweight or obese), 25.0 to 29.9 kg/m^2^ (as overweight), and ≥ 30.0 kg/m^2^ (as obese) [29]. The fasting blood sugar and blood cholesterol levels were determined using the Cardiocheck Plus Analyzer (PTS Diagnostics, Indianapolis, USA), based on blood samples obtained by the fingerstick method according to the WHO STEPS manual [30]. The participants were instructed to fast for at least 12 hours before the blood samples were taken. Diabetes was diagnosed if the fasting blood sugar level was 126 mg/dL or higher or the participants were taking any anti-diabetic medications at the time of the interview [31]. The cut-off value for the high cholesterol level was ≥ 240 mg/dL [32].

### Data analysis

We analysed the data using the STATA software version 16.0 (Stata Corporation, College Station, TX, USA). All estimations were weighted using the population weights to account for the complex survey design and were presented together with their 95% confidence intervals (CIs).

Gaps in the cascade of hypertension care were presented in the flow diagram that represents the percentages of people who took part in and left each step of care. To calculate the percentage of people taking part in each step, the denominator was held constant throughout the sequential steps, so that the cumulative losses in the cascade of care were visible. The differences in the prevalence of hypertension screening, awareness, treatment, and control by survey years were tested using the chi-square test.

To assess the degree of socio-economic inequalities in the cascade of hypertension care, we plotted a concentration curve using the cumulative percentage of each indicator (*y*-axis) against the cumulative percentage of wealth quintiles (*x*-axis). We estimated the concentration index for each indicator. Given the dichotomous nature of the outcome variables, we employed Erreygers Corrected Concentration Index and specified the limits as 0 and 1 [33].

We conducted a Poisson regression analysis to report the prevalence ratio, to allow for a straightforward interpretation of the data, and to account for the low prevalence of hypertension treatment and control [34]. We included all available explanatory variables in the multivariable models (as a model I). All of the models were adjusted for the survey year. As a sensitivity analysis, we also conducted a subgroup analysis as a model II for hypertension treatment (among the aware hypertensives only) and hypertension control (among the treated hypertensives only); the results are shown in Supplementary file 1.

## Results

### Characteristics of the participants

The majority of the hypertensive participants (57.9%) were men. The mean (standard deviation) age of the participants was 40.2 years (14 years). Most of the participants (84.9%) were married. Nearly half of the participants (48.8%) had received no formal schooling or had not completed the primary level of education. Around three-fourths of the participants (75.7%) were either homemakers or self-employed. Only 3% and 7.4% of the participants consumed the recommended amounts of fruit and vegetables, respectively. A vast majority of participants (93.7%) engaged in the recommended level of physical activity per week. Of the hypertensive participants, 9% also had diabetes and 5.9% also had high cholesterol levels (Table 1).

**Table 1.** Characteristics of hypertensive participants

Variables	*n*	%*
**Age**		
15–29 years	296	24.2
30–44 years	851	28.9
45–69 years	1645	46.9
**Sex**		
Men	1236	57.9
Women	1556	42.1
**Marital status**		
Never married	123	10.2
Currently married	2451	84.9
Widowed	190	4.2
Other (separated, divorced)	27	0.6
**Education**		
No formal schooling	1305	37.7
Lower than primary school	328	11.1
Primary school	421	17.5
Secondary school	454	20.7
High school	174	8.3
Bachelor’s degree and higher	109	4.8
**Occupation**		
Government employee	86	3.1
Non-government employee	170	8.1
Self-employed	788	33.2
Homemaker	1510	42.5
Student	61	5.3
Unemployed	63	3.3
Other (retired, non-paid job)	124	4.4
**Smoking**		
Yes^†^	610	22.0
No	2182	78.0
**Alcohol consumption**		
Yes^‡^	714	27.3
No	2078	72.7
**Vegetable intake**		
Sufficient^§^	196	7.4
Insufficient	2596	92.6
**Fruit intake**		
Sufficient^∥^	87	3.0
Insufficient	2705	97.0
**Physical activity**		
Sufficient^¶^	2575	93.7
Insufficient	184	6.3
**Body mass index**		
< 25 kg/m^2^	1718	63.9
25–29 kg/m^2^	799	27.8
≥ 30 kg/m^2^	261	8.3
**Diabetes**		
Yes**	263	9.0
No	2348	91.0
**Cholesterol level**		
High (≥ 240 mg/dL)	177	5.9
Not high	2470	94.1

Note: *weighted percentage ^†^Smoking tobacco at least once in the 30 days prior to the survey; ^‡^At least one drink of alcohol in the 30 days prior to the survey; ^§^Eating at least three servings of vegetables in a typical week; ^∥^Eating at least two servings of fruit in a typical week; ^¶^Involvement in moderate and/or vigorous physical activity of ≥ 600 MET minutes/week in a week; **Fasting blood sugar level of 126 mg/dL or higher or taking any anti-diabetic medications at the time of the interview.

### Gaps in the cascade of hypertension care

**Figure 1.**
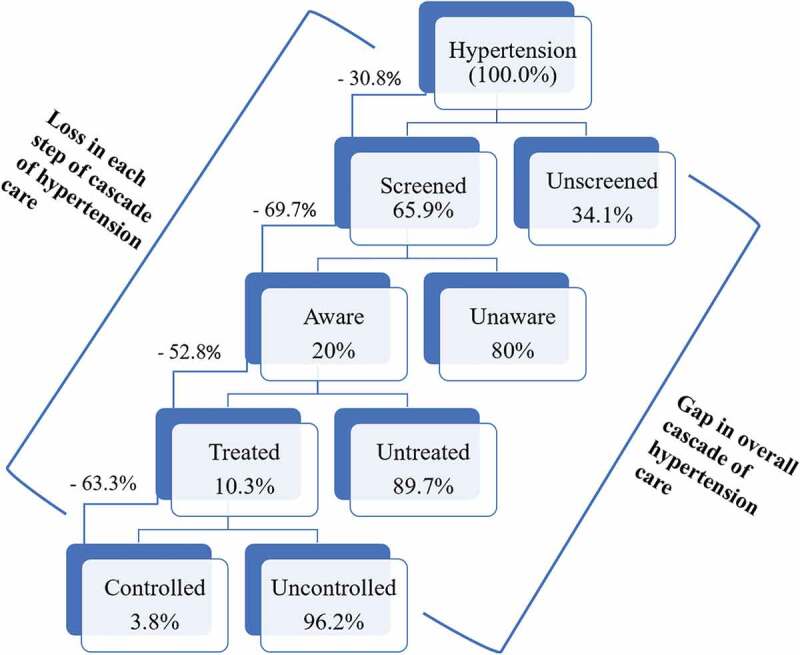
Gaps in the cascade of hypertension care.

Note: The denominator for the overall loss in the cascade of care is the total number of hypertensive participants (*n* = 2792). The denominator for the gap at each step in the cascade of hypertension care is the number of the participants from the antecedent step. For example, the denominator for the loss in treatment is the total number of aware hypertensive participants (*n* = 682)

The prevalence of hypertension was 25.4% (95% CI: 23.9, 27.0). Among the hypertensive participants, the prevalence of hypertension screening was 65.9% (95% CI: 62.2, 69.5), the prevalence of hypertension awareness was 20% (95% CI: 18.1, 22.1), the prevalence of hypertension treatment was 10.3% (95% CI: 8.8, 12), and the prevalence of hypertension control was 3.8% (95% CI: 2.9, 4.9). In the cascade of care, 34.1% (95% CI: 30.5, 37.8) of the hypertensive participants did not have their blood pressure screened. Of those screened, 30.3% (95% CI: 27.6, 33.1) were aware of their hypertension (Figure 1). Among aware hypertensives, less than half (47.2%; 95% CI: 41.9, 52.6) were receiving treatment. Of those who were receiving treatment, 36.7% (95% CI 30.1, 43.8) had controlled high blood pressure.

### Trend in the cascade of hypertension care

The prevalence of awareness (19.7% vs 20.3%), treatment (11.7% vs 9.0%), and control (3.8% vs 3.8%) did not differ significantly (*p* > 0.05 for all) between the 2013 and 2019 surveys. The difference in the prevalence of hypertension screening between the two survey years (70.3% vs 61.8%) was significant (*p* = 0.036).

### Socio-demographic variation in the cascade of hypertension care

Unadjusted prevalence estimates indicated that hypertension screening (*p* < 0.001), awareness (*p* < 0.001), treatment(*p* < 0.001), and control (*p* < 0.013) significantly varied across the age categories (Table 2). The prevalence of hypertension awareness was significantly lower among men as compared to women (17.2% vs 23.9%, *p* < 0.001). Hypertension screening (*p* = 0.035), awareness (*p* < 0.001), and treatment (*p* = 0.003) varied significantly across the groups by marital status. The lowest prevalence of hypertension screening (54.4%), awareness (6.7%), and treatment (2.7%) was found among those who had never been married. The lowest prevalence of screening was found among those who had had no formal education. The prevalence of hypertension awareness (*p* < 0.001) and control (*p* < 0.001) varied significantly across the occupational groups, with the lowest prevalence found among students.

**Table 2.** Sociodemographic distribution of screening, awareness, treatment, and control of hypertension among people with hypertension in Nepal

	Screened	Aware	Treated	Controlled
Variables	%	%	%	%
**Age**				
15–29 years	52.0	6.2	2.2	1.5
30–44 years	70.3	17.9	7.0	3.3
45–69 years	70.5	28.4	16.5	5.2
*p-*value*	< 0.001	< 0.001	< 0.001	0.013
**Sex**				
Men	64.9	17.2	9.3	3.1
Women	67.3	23.9	11.7	4.7
*p-*value*	0.407	< 0.001	0.105	0.105
**Marital status**				
Never married	54.4	6.7	2.7	2.5
Currently married	67.8	21.4	11.0	3.9
Widowed	56.1	25.1	13.7	5.4
Other (separated, divorced)	64.1	20.9	20.9	0.0
*p-*value*	0.035	< 0.001	0.003	0.595
Education				
No formal schooling	59.6	21.7	12.0	3.8
Lower than primary school	67.8	21.7	7.5	2.7
Primary school	69.9	20.5	9.2	3.6
Secondary school	67.6	15.9	8.4	4.3
High school	66.3	17.7	9.9	2.8
Bachelor’s degree and higher	88.9	23.4	16.5	6.4
*p-*value*	0.004	0.303	0.083	0.706
Occupation				
Government employee	78.6	28.1	18.8	7.9
Non-government employee	61.8	16.5	5.5	0.3
Self-employed	66.5	19.4	10.0	4.2
Homemaker	66.0	21.0	10.6	4.0
Student	55.2	4.1	2.1	2.1
Unemployed	59.1	8.3	3.6	3.3
Other (retired, non-paid job)	77.5	44.2	27.6	4.2
p-value*	0.344	< 0.001	< 0.001	0.306

Note: * p-value from chi-square test

#### Geographical variation in the cascade of hypertension care

**Figure 2.**
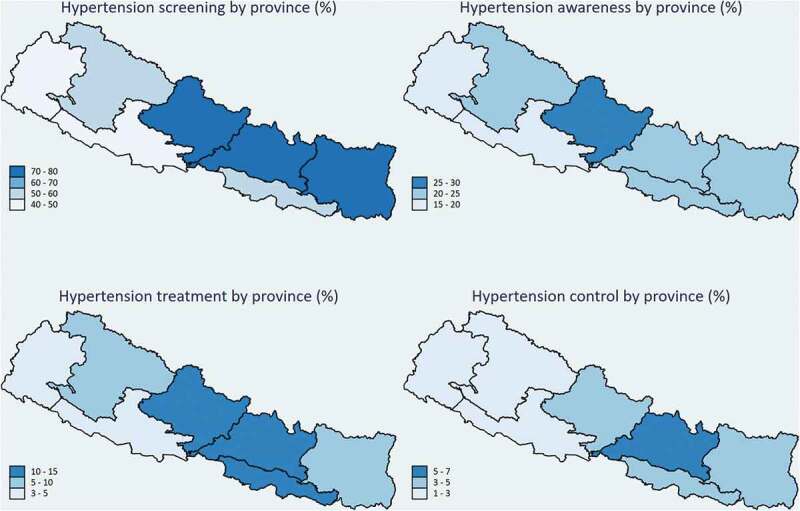
Geographical variation in the cascade of hypertension care.

Note: From right to left, the provinces are named as Province 1, Province 2, Bagmati, Gandaki Lumbini, Karnali, and Sudurpaschim, separated by a black border (line)

Gandaki Province had the highest prevalence of hypertension screening (76.3%) and hypertension awareness (26.4%) among the seven provinces (Supplementary file 2). Bagmati Province had the highest percentage of participants being treated (13.0%) and having optimal control of hypertension (7.1%). Hypertension screening, awareness, treatment, and control were relatively low in the Lumbini and Sudurpaschim provinces (Figure 2). We did not find a significant difference between rural and urban settings in the cascade of hypertension care.

### Socio-economic inequalities in the cascade of hypertension care

**Figure 3.**
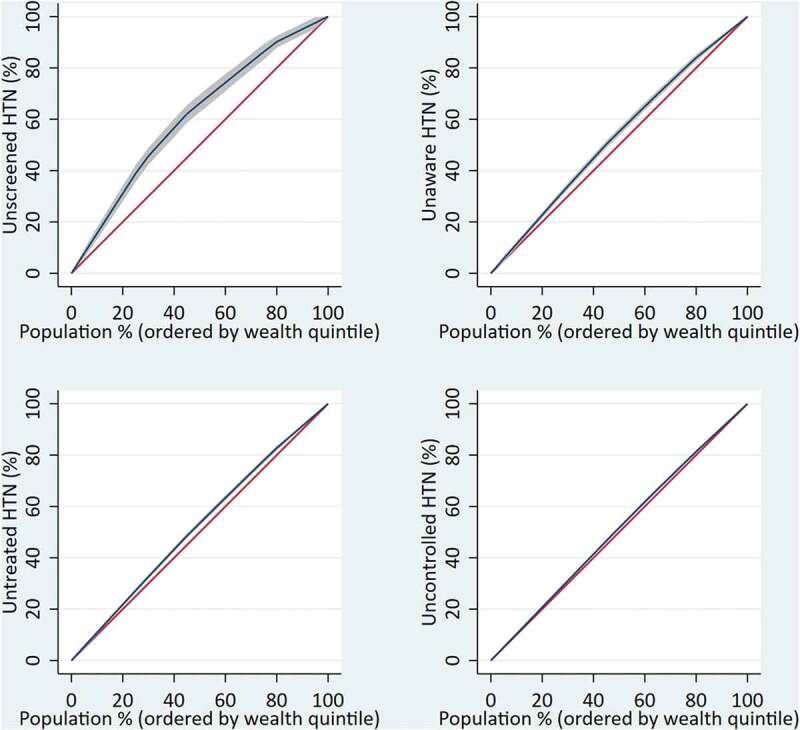
Economic inequalities in the cascade of hypertension care.

Note: *y*-axis is the cumulative percentage of the outcome variables, *x*-axis is the cumulative percentage of population ordered by the wealth quintile from the lowest to the higher quintile; HTN = hypertension. The concentration index (cin) is defined as twice the area between the concentration curve (blue) and the line of equality (the 45-degree red line). A positive cin (curve below the line of equality) indicates that ill/undesired health is more prevalent among the rich, and a negative one (curve above the line of equality) indicates that the outcome is more prevalent among the poor.

Hypertension screening, awareness, treatment, and control were significantly associated with the wealth quintile (Supplementary file 2). The probability of being screened, aware, treated, and controlled for hypertension increased as the wealth quintile increased. The undesired outcomes – unscreened (concentration index [cin] = −0.19), unaware (cin = −0.16), untreated (cin = −0.11), and uncontrolled (cin = −0.06) hypertension) – were the highest among the poorest Nepalese (Figure 3).

### Gaps in the cascade of hypertension care by healthcare providers and financing

**Figure 4.**
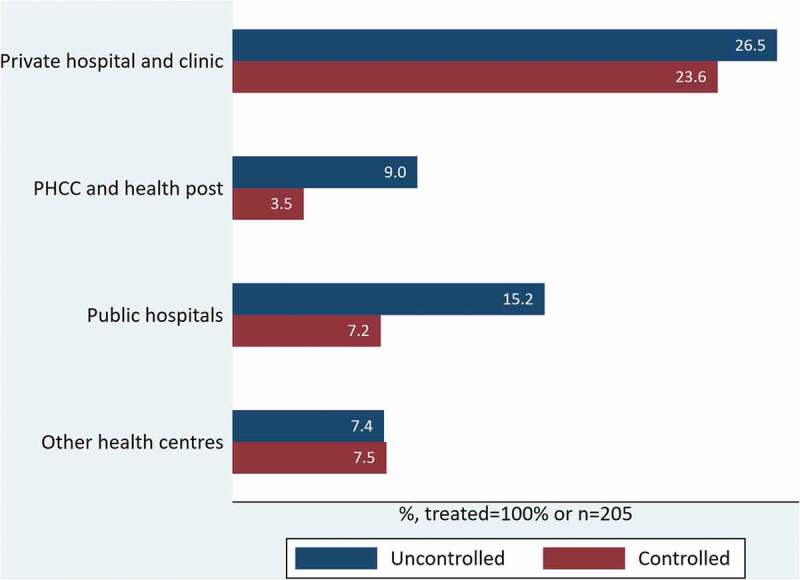
Hypertension treatment and control by the providers.

Note: PHCC – Primary healthcare centres; Other health centres – community hospitals, Ayurveda health centres and pharmacies

Of the participants receiving medication, around half (50.2%) had sought treatment in private health centres, 34.9% had visited public hospitals and primary healthcare centres, and 14.9% had received treatment from other intuitions, such as community health centres, Ayurveda hospitals (i.e. Ayurveda health centres functioning at the district level under the Ministry of Health), and pharmacies (i.e. chemist shops) (Figure 4). The hypertension control rate was higher among those who had been treated in private health institutions (50.4%) as compared with those who had sought treatment in primary healthcare centres (27.8%) and public hospitals (32.2%) (Figure 4)

### Factors associated with the cascade of hypertension care

#### Factors associated with hypertension screening

The probability of being screened was positively associated with the age, education level, and body mass index. Screening was more prevalent in the people with diabetes, those who did not consume alcoholic drinks, and those who ate more fruits (Table 3).

**Table 3.** Factors associated with hypertension screening, awareness, treatment, and control

	Adjusted prevalence ratio (95% CI*)			
Variables	Screening	Awareness	Treatment	Control
**Age**				
15–29 years	Ref	Ref	Ref	Ref
30–44 years	1.34 (1.13, 1.58)	2.14 (1.22, 3.77)	3.22 (1.16, 8.95)	4.43 (0.71, 27.64)
45–69 years	1.41 (1.20, 1.67)	3.51 (2.06, 6.00)	7.25 (2.65, 19.86)	Sex7.35 (1.14, 47.44)
**Sex**				
Men	Ref	Ref	Ref	Ref
Women	1.08 (0.97, 1.21)	1.65 (1.30, 2.10)	1.26 (0.87, 1.82)	1.62 (0.86, 3.03)
**Marital status**				
Never married	Ref	Ref	Ref	Ref
Currently married	1.0 (0.77, 1.30)	0.95 (0.46, 1.95)	0.94 (0.32, 2.72)	0.38 (0.10, 1.47)
Widowed	0.85 (0.63, 1.16)	0.85 (0.39, 1.84)	0.83 (0.28, 2.47)	0.48 (0.13, 1.83)
Other (separated, divorced)	0.95 (0.56, 1.62)	0.82 (0.29, 2.32)	1.96 (0.51, 7.57)	
Education				
No formal schooling	Ref	Ref	Ref	Ref
Lower than primary school	1.19 (1.06, 1.35)	1.15 (0.86, 1.55)	0.73 (0.48, 1.12)	0.96 (0.44, 2.11)
Primary school	1.29 (1.15, 1.45)	1.30 (0.98, 1.72)	1.08 (0.72, 1.63)	1.70 (0.85, 3.38)
Secondary school	1.27 (1.12, 1.44)	1.14 (0.85, 1.54)	1.09 (0.68, 1.75)	2.09 90.98, 4.44)
High school	1.24 (1.02, 1.51)	1.38 (0.85, 2.25)	1.28 (0.68, 2.41)	1.48 0.43, 5.16)
Bachelor’s degree and higher	1.51(1.24, 1.83)	1.36 (0.88, 2.09)	1.48 (0.78, 2.82)	2.57 (0.81, 8.11)
Occupation				
Government employee	Ref	Ref	Ref	Ref
Non-government employee	0.97 (0.76, 1.25)	0.89 (0.48, 1.64)	0.58 (0.25, 1.38)	0.07 (0.01, 0.39)
Self-employed	0.99 (0.81, 1.20)	0.86 (0.49, 1.50)	0.77 (0.35, 1.69)	0.73 (0.17, 3.03)
Homemaker	0.98 (0.80, 1.21)	0.69 (0.39, 1.21)	0.66 (0.30, 1.46)	0.59 (0.14, 2.52)
Student	0.95 (0.61, 1.50)	0.41 (0.08, 2.05)	0.68 (0.09, 5.24)	0.73 (0.08, 6.85)
Unemployed	0.92 (0.66, 1.29)	0.38 (0.12, 1.22)	0.34 (0.05, 2.30)	0.65 (0.07, 6.17)
Other (retired, non-paid job)	1.05 (0.85, 1.31)	1.34 (0.74, 2.43)	1.12 (0.50, 2.51)	0.42 0.10, 1.78)
Smoking				
Yes^†^	Ref	Ref	Ref	Ref
No	0.99 (0.90, 1.09)	0.93 (0.74, 1.18)	1.0 (0.70, 1.43)	0.72 (0.40, 1.27)
Alcohol consumption				
Yes^‡^	Ref	Ref	Ref	Ref
No	1.10 (1.00, 1.22)	0.99 (0.78, 1.25)	1.56 (1.05, 2.31)	2.59 (1.08, 6.22)
Vegetable intake				
Sufficient^∥^	Ref	Ref	Ref	Ref
Insufficient	0.99 (0.89, 1.11)	0.98 (0.70, 1.38)	1.28 (0.79, 2.06)	1.13 (0.53, 2.44)
Fruit intake				
Sufficient^∥^	Ref	Ref	Ref	Ref
Insufficient	0.90 (0.80, 1.03)	0.68 (0.48, 0.97)	0.63 90.38, 1.05)	0.57 (0.23, 1.41)
Physical activity				
Sufficient^¶^	Ref	Ref	Ref	Ref
Insufficient	0.95 (0.84, 1.07)	1.51 (1.14, 1.99)	1.57 (1.04, 2.36)	1.82 (0.96, 3.45)
Body mass index				
< 25 kg/m2	Ref	Ref	Ref	Ref
25–29 kg/m2	1.12 (1.02, 1.22)	1.54 (1.26, 1.88)	1.82 (1.36, 2.43)	1.68 (1.00, 2.83)
≥ 30 kg/m2	1.33 (1.21, 1.47)	1.98 (1.51, 2.59)	2.18 (1.48, 3.20)	2.31 (1.20, 4.46)
Diabetes				
Yes**	Ref	Ref	Ref	Ref
No	0.89 (0.81, 0.97)	0.68 (0.55, 0.85)	0.58 (0.43, 0.79)	0.82 (0.45, 1.50)
Cholesterol level				
High (>239 mg/dL)	Ref	Ref	Ref	Ref
Not high	1.06 (0.93, 1.22)	0.80 (0.57, 1.11)	0.74 (0.49, 1.10)	0.56 (0.28, 1.11)

Note: *Prevalence ratio adjusted for all the remaining variables listed in the table, survey year, and responses to a question that combines ethnicity, historical caste groups, religion, and social disadvantage and its 95% Confidence interval; ^†^Smoking tobacco at least once in the 30 days prior to the survey; ^‡^At least one drink of alcohol in the 30 days prior to the survey; ^∥^Eating at least three servings of vegetables in a typical week; ^∥^Eating at least two servings of fruit in a typical week; ^¶^Involvement in moderate and/or vigorous physical activity of ≥ 600 MET minutes/week in a week; **Fasting blood sugar level of 126 mg/dL or higher or taking any anti-diabetic medications at the time of the interview.

#### Factors associated with hypertension awareness

Age was positively associated with the hypertension awareness. More men than women were aware of their hypertension. Less physically active, overweight, and obese participants had a higher prevalence of awareness than others (Table 3).

#### Factors associated with hypertension treatment

The probability of getting treatment was three and seven times higher among the groups of participants who were 30–44 years and 45–69 years of age as compared to the 15–29-year-old hypertensive participants. We found no significant association with gender in the whole sample. However, while considering only the participants who were aware of their hypertension, the prevalence of hypertension treatment among women was 28% lower than among men (*p* < 0.021); Supplementary file 1). The treatment rate was significantly higher among obese and diabetic participants and those who did not consume alcoholic drinks (Table 3).

#### Factors associated with hypertension control

The prevalence of hypertension control in the 45-years-and-above age group was six times higher than that of 15–29-year-old participants. The rate of hypertension control did not vary significantly by gender, marital status, and education. Compared to government employees, the prevalence of hypertensive participants with controlled blood pressure was significantly lower among those working in non-government sectors. The hypertension control rate was 2.59 times higher in hypertensives who did not drink alcohol than among those who drank alcohol. The probability of having controlled blood pressure increased as the age increased (Table 3).

## Discussion

This study found a low prevalence of screening, awareness, treatment, and control of hypertension in Nepal, indicating large gaps in the cascade of hypertension care. Only 3.8% of the participants were found to have controlled blood pressure. The cases of unscreened, unaware, untreated, and uncontrolled hypertension were more prevalent amongst the poorer participants, those living in the Lumbini and Sudurpaschim provinces, those who had sought treatment in primary healthcare centres and public hospitals, those who had no health insurance, and in younger age groups. These findings should facilitate the revision of the existing hypertension care strategies and reallocation of the existing resources to achieve a better control of blood pressure among hypertensive individuals in Nepal.

The prevalence estimates for hypertension awareness, treatment, and control found in the current study are the lowest ever reported in Nepal. The prevalence estimates reported in four previous studies conducted in different parts of Nepal ranged from 43% to 61.8% for hypertension awareness, from 29.0% to 48.7% for hypertension treatment, and from 8.2% to 24.1% for hypertension control [35–38]. The prevalence estimates from the Nepalese Demographic Health Survey on awareness (40.0%), treatment (19.2%), and control (10.5%) were also higher than those found in the current study [39]. The reason for such differences in the estimates may be due to the differences in the study populations across the studies. For example, the participants in the Dhungana et al. [8] and Karmacharya et al. [12] studies were from Bagmati province only, where – as our study findings suggest – the prevalence of hypertension control is higher than in the other provinces. Likewise, the Nepalese Demographic Health Survey also included participants aged 70 years and above [39]. Given the fact that the sample in the current study was restricted to adults aged 15–69 years, direct comparisons between our findings and those of the Nepalese Demographic Health survey would not be justified. As suggested by our results, the prevalence of hypertension awareness, treatment, and control is higher in older age groups. It is, therefore, not surprising that the prevalence estimates from the Nepalese Demographic Survey are higher than those we found.

The study findings also suggest that Nepal has the poorest performance in the cascade of hypertension care as compared with the neighbouring countries. For example, hypertension control rates in India [16] and China [40] are nearly twice as high as those in Nepal.

In comparison, USA (53%) and Canada (66%) have the highest prevalence of hypertension control and are examples of countries that provide effective hypertension care [41]. After introducing the Canadian Hypertension Education Program, Canada was able to improve hypertension treatment from 35% to 80% and hypertension control from 13% to 68% between 1992 and 2013 [42]. Some strategies used in this program might also be applicable to the Nepalese context.

The gaps in the cascade of hypertension care were inversely related to wealth. A higher prevalence of hypertension screening, awareness, treatment, and control was associated with higher wealth quintiles. The socio-economic inequalities in health and healthcare utilization are common in low- and middle-income countries [43–45]. A study conducted among 163,397 participants from 21 countries found that better economic development (as measured as gross national product (GNP) per capita) of the countries and higher socio-economic status (as expressed in wealth quintiles) of the individuals were positively associated with awareness, treatment, and control of hypertension [46]. Based on these findings, the lower rate of treatment and control in the Lumbini and Sudurpaschim provinces could also be explained by their geographical remoteness and high poverty rates.

Our study also found that the prevalence of hypertension treatment and control was significantly higher in people who had health insurance coverage. Previous studies showed that patients with health insurance are less likely to report barriers in accessing hypertension care [47] and achieve greater reductions in blood pressure than uninsured persons [48]. These findings suggest that improving the accessibility of health insurance may positively affect hypertension care in Nepal. However, further studies are required to evaluate and confirm the benefit of the current health insurance policy in terms of improving access to healthcare and disease control. Likewise, the association observed between primary healthcare and government hospitals and a poor control of hypertension indicates a need to improve the quality of services at these institutions, which would also help minimize the socio-economic inequalities in hypertension care [49].

An age disparity in the cascade of hypertension care was prominent. Participants in the lower age group (< 30 years) were less likely to be screened, aware, treated, and have controlled blood pressure, results that are consistent with those of American studies that found that young adults had a 33% lower rate of being diagnosed [50] and a 44% lower rate of medication initiation [51]. The literature shows that young adults think hypertension develops during old age and that taking medication makes them feel older [52]. Hypertension treatment and control did not vary significantly across gender, marital status, education, and occupation groups. However, the probability of being enrolled in antihypertensive treatment was higher for men than for women, if they were aware that they had hypertension.

Except for alcohol consumption, other behavioural risk factors such as smoking, fruits and vegetable intake, and physical activity were not significantly associated with hypertension control. Those who did not drink alcohol were more likely to take medication and have controlled blood pressure. Participants with a higher BMI and those with diabetes were more likely to seek hypertension treatment than others. However, hypertension control was not associated with diabetes. Although most of the study variables were not significantly associated with hypertension control, it is important to note that all the participants who had controlled blood pressure had also been taking antihypertensive drugs. However, studies have shown that several barriers to hypertension treatment exist in Nepal that potentially impede the initiation of treatment and adherence and lead to uncontrolled blood pressure among hypertensive patients [53,54]. Therefore, along with the interventions to reduce exposure to risk factors, it is prudent to develop strategies that can dismantle the barriers associated with hypertension treatment and control in order to achieve the target of a relative 25% reduction in hypertension by 2025 in Nepal [55].

This study had some limitations. The surveys were not primarily designed to assess gaps in the cascade of hypertension care. Therefore, this study lacked some potentially important explanatory variables, such as medication adherence. Furthermore, the findings on fruits and vegetable intake, physical activity, smoking, and alcohol consumption might have been influenced by the recall and social desirability biases, as the responses were collected via self-reports. In addition, the blood pressure was measured on a single occasion only, which may have resulted in a miscategorization of some participants. Similarly, the finding is limited to the quantitative assessment of the gaps. Further qualitative studies are required to gain a deeper understanding of the contextual factors (e.g. perceived barriers and facilitators) that are likely to be associated with the gaps in the cascade of hypertension care in Nepal.

The main strength of the study is the representative nature of the data we used for the analysis. STEPS surveys follow the standard framework and methods of the WHO STEPwise Approach to Noncommunicable Disease Risk-Factor Surveillance to collect nationally representative data. Furthermore, this study represents an original contribution to the knowledge base in that it quantifies the gap in hypertension care and depicts its distribution across different population groups, particularly because a large variety of participant characteristics were taken into account.

## Conclusions

The gaps in the cascade of hypertension care in Nepal are large, and the rate of hypertension control is critically low. The gaps are particularly pronounced among the poor, those living in Lumbini and Sudurpaschim provinces, those who had sought treatment in primary healthcare centres and public hospitals, those who did not have health insurance, and young people. National- and local-level public health interventions are needed to improve hypertension screening, awareness, treatment, and control in Nepal. Mass screening that targets the most heavily affected areas and population groups, increasing access to quality care services at public primary healthcare centres and public hospitals, and applying behavioural interventions to address the barriers to hypertension treatment and control are recommended. Expanding the role of community health workers in supporting hypertension management and medication adherence could be a feasible strategy to help patients overcome barriers to hypertension treatment and control in the Nepalese context.

## Data Availability

Data used in the study are available from the Nepal Health Research Council upon a reasonable request.

## Paper context

Quantifying the gaps in the cascade of hypertension care is critical for improving management of hypertension. This study assessed the prevalence of hypertension screening, awareness, treatment, and control in Nepal and associated factors. The findings may inform public health stakeholders in Nepal about losses of hypertensive participants that occur in each step of the cascade of hypertension care and about the population groups that are at the highest risk of poor hypertension care in Nepal.
